# Leaf Photosynthetic and Functional Traits of Grassland Dominant Species in Response to Nutrient Addition on the Chinese Loess Plateau

**DOI:** 10.3390/plants11212921

**Published:** 2022-10-30

**Authors:** Yuan Jin, Shuaibin Lai, Zhifei Chen, Chunxia Jian, Junjie Zhou, Furong Niu, Bingcheng Xu

**Affiliations:** 1 State Key Laboratory of Soil Erosion and Dryland Farming on the Loess Plateau, Northwest A&F University, Xianyang 712100, China; 2 College of Life Sciences, Guizhou University, Guiyang 550025, China; 3 College of Forestry, Gansu Agricultural University, Lanzhou 730070, China; 4 Institute of Soil and Water Conservation, Chinese Academy of Sciences and Ministry of Water Resources, Xianyang 712100, China

**Keywords:** semiarid grassland, fertilization, leaf functional trait, leaf photosynthesis, atmospheric nitrogen deposition

## Abstract

Leaf photosynthetic and functional traits of dominant species are important for understanding grassland community dynamics under imbalanced nitrogen (N) and phosphorus (P) inputs. Here, the effects of N (N0, N50, and N100, corresponding to 0, 50, and 100 kg ha^−1^ yr^−1^, respectively) or/and P additions (P0, P40, and P80, corresponding to 0, 40, and 80 kg ha^–1^ yr^–1^) on photosynthetic characteristics and leaf economic traits of three dominant species (two grasses: *Bothriochloa ischaemum* and *Stipa bungeana*; a leguminous subshrub: *Lespedeza davurica*) were investigated in a semiarid grassland community on the Loess Plateau of China. Results showed that, after a three-year N addition, all three species had higher specific leaf area (SLA), leaf chlorophyll content (SPAD value), maximum net photosynthetic rate (*P*_Nmax_), and leaf instantaneous water use efficiency (WUE), while also having a lower leaf dry matter content (LDMC). The two grasses, *B. ischaemum* and *S. bungeana*, showed greater increases in *P*_Nmax_ and SLA than the subshrub *L. davurica*. P addition alone had no noticeable effect on the *P*_Nmax_ of the two grasses while it significantly increased the *P*_Nmax_ of *L. davurica*. There was an evident synergetic effect of the addition of N and P combined on photosynthetic traits and most leaf economic traits in the three species. All species had relatively high *P*_Nmax_ and SLA under the addition of N50 combined with P40. Overall, this study suggests that N and P addition shifted leaf economic traits towards a greater light harvesting ability and, thus, elevated photosynthesis in the three dominant species of a semiarid grassland community, and this was achieved by species–specific responses in leaf functional traits. These results may provide insights into grassland restoration and the assessment of community development in the context of atmospheric N deposition and intensive agricultural fertilization.

## 1. Introduction

The semiarid Loess Plateau region in China is characterized by severe water scarcity, soil erosion, and nutrient-poor soils, which all greatly limit vegetation growth [1]. Grassland, with a size of ~2.7 × 10^5^ km^2^, accounts for ca. 43% of the regional total land area and is the dominant vegetation type on the Plateau [2]. It provides essential ecosystem functions and services such as carbon sequestration, soil and water conservation, and biodiversity [3,4]. Grassland management and restoration are of great ecological and economic significance in the region. Fertilization, as an effective management practice to increase grassland productivity and promote grassland restoration, is adopted in many natural/semi-natural grasslands, e.g., in the Inner Mongolian steppe [5], the alpine meadow on the Qinghai–Tibet Plateau [6] in China, and in the European alpine grasslands [7] and the North American Great Plains [8]. However, fertilization may also lead to undesirable consequences such as exacerbated eutrophication [9], reduced ecosystem resilience (e.g., increased grassland drought sensitivity [8,10]), and biodiversity loss [11,12,13], which subsequently alter community composition and structure, as well as decrease the effects of diversity stability on maintaining grassland productivity [14]. Soils in the semiarid Loess Plateau are commonly deficient in both nitrogen (N) and phosphorus (P) [15]. Previous studies in the region have shown that appropriate N and P fertilization could improve soil N and P availability and play a positive role in increasing grassland productivity and recovery of degraded grasslands [16,17,18]. On the other hand, the effects of the N and P addition on photosynthetic and leaf functional traits of dominant species, which are important for understanding underlying mechanisms of grassland community dynamics under N and P inputs, have only received limited attention in the regional grassland.

Plant photosynthesis is the basis of plant growth, and its diurnal patterns reflect the sustained carbon assimilation ability of plants and have been extensively studied across a wide range of arid and semiarid grassland species, particularly in North America (e.g., [19,20,21,22]). Previous studies have explored the photosynthetic diurnal dynamics—under elevated CO_2_ conditions—in dry and wet years [22,23], the diurnal photosynthetic performance of grasses with contrasting functional types (e.g., C_3_ vs. C_4_, invasive vs. native) [20,21], and biotic and abiotic controls of photosynthetic diurnal courses [19,24]. However, there is limited information on the impacts of nutrient addition on photosynthetic diurnal courses in dryland grassland species. As an essential element of all proteins in plants (e.g., nucleic acid, enzymes, and chlorophyll), N primarily determines plant photosynthetic performance [25,26]. Extensive studies have documented that N addition increases plant photosynthetic rate and promotes plant growth in grasslands [27,28], and this promotion may be mediated by soil moisture [10]. However, when N addition exceeds a threshold, it will not continue to increase plant photosynthesis or, even, inhibit plant growth [26,29]. P, as another essential macronutrient, is also vital for plant photosynthesis, and it is the main component of ATP, NADPH, and phospholipids, which all play important roles in regulating photosynthesis machinery and electron transport activities [30,31]. Apart from the direct regulation of N/P on plant photosynthesis, N and P addition could indirectly or interactively affect plant photosynthesis. For instance, P addition could improve photosynthesis by increasing leaf area and stomatal aperture, particularly under soil water deficit conditions [32,33]. P addition could increase the activity of N-fixing bacteria, nodule biomass, and nitrogenase activity in legumes, which subsequently increases leaf N and P content and photosynthetic rate [34]. The combined fertilization of N and P is, thereby, often considered an effective management tool for sustaining productivity in many grassland communities, while such effects need to be evaluated in the regional semiarid grassland on the Loess Plateau.

Besides photosynthetic characteristics, other important leaf functional traits, such as specific leaf area (SLA), leaf dry matter content (LDMC), leaf nitrogen mass (*N*_mass_), and leaf phosphorus mass (*P*_mass_), are also the intuitive representation of strategies adopted by plants to cope with environmental changes [35,36]. According to the leaf economic spectrum (LES) theory [37], angiosperm plants could generally be divided into a rapid/slow growing strategy according to a set of leaf functional traits, with the rapid-growing ones having low LDMC and high *P*_n_, SLA, *N*_mass_, and *P*_mass_; contrarily, the slow-growing ones have the opposite leaf traits. This kind of functional trait-based theory, from leaf to plant levels, provides great insights into understanding species resource utilization and species distribution [37]. Nevertheless, species-specific responses in these leaf economic traits exist under varied environmental (e.g., under different soil water and nutrient availability) conditions, which should be systematically assessed [38]. Efforts have been made to quantify the variation of LES of grassland species under differed soil nutrient availability, which confirmed species-specific patterns [39], while the assessment of species-specific responses to varying N and P fertilization conditions is seldom conducted on the semiarid Loess Plateau, which is needed to better understand grassland community development.

The dominant species occupy important ecological niches and play vital roles in maintaining community structure and function [40,41]. Biomass increases after N/P additions tend to be achieved by decreasing species diversity or increasing the biomass of dominant species [42]. Quantifying physiological and growth characteristics of dominant species under N and/or P addition could, thereby, be important for the evaluation of community productivity and dynamics, as well as provide valuable information for grassland management and restoration. *Bothriochloa ischaemum* (L.) Keng (a C_4_ perennial grass), *Stipa bungeana* Trin. (a C_3_ perennial grass), and *Lespedeza davurica* (Laxm.) Schindl. (a C_3_ N-fixing subshrub) are co-dominant species in the natural/restored grasslands on the semiarid Loess Plateau of China [43]. The previous study on a regional grassland community, targeting these species, has shown that the addition of N and P combined improved grassland productivity and decreased species diversity, primarily via effects on tall clonal and annual species [44], which, once again, suggested species or functional-type-specific responses within a community, while the variation of leaf functional traits in these dominant species, after N and P fertilization, have not been fully assessed. Thus, we examined photosynthetic diurnal change, SPAD value, and leaf economic traits, including *N*_mass_, *P*_mass_*, N_mass_/P_mass_* ratio, SLA, and LDMC of the three dominant species, following a three-year N and P addition experiment in a typical semiarid grassland community on the Loess Plateau. We tested the hypotheses that: (1) N/P addition would increase photosynthetic rates and alter the photosynthetic diurnal dynamics of the three dominant species in the peak growing season, and these photosynthetic responses would be related to species-specific shifts in leaf functional traits; (2) addition of N and P combined would further promote photosynthesis compared with N/P additions alone.

## 2. Results

### 2.1. Environmental Factors

**Figure 1:**
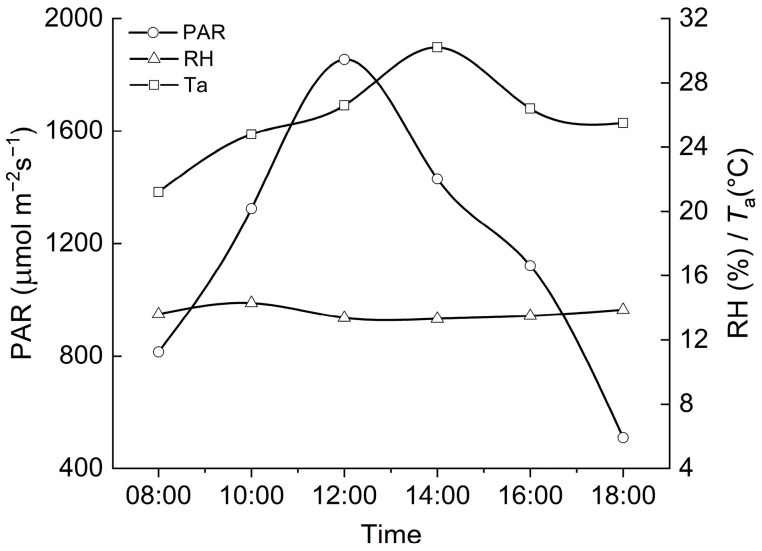
Diurnal changes of photosynthetically active radiation (PAR), air relative humidity (RH), and air temperature (*T*_a_) during the measurement period (20–22 July 2019).

Photosynthetically active radiation (PAR) and air temperature (*T*_a_) showed a single-peaked diurnal curve during the experimental period, and the maximum values appeared at 12:00 h and 14:00 h, with the values of 1854 μmol·m^−2^·s^−1^ and 30.2 °C, respectively (Figure 1). The relative humidity (RH) remained stable during the daytime (~13%) (Figure 1).

### 2.2. Diurnal Changes in Photosynthesis

**Figure 2:**
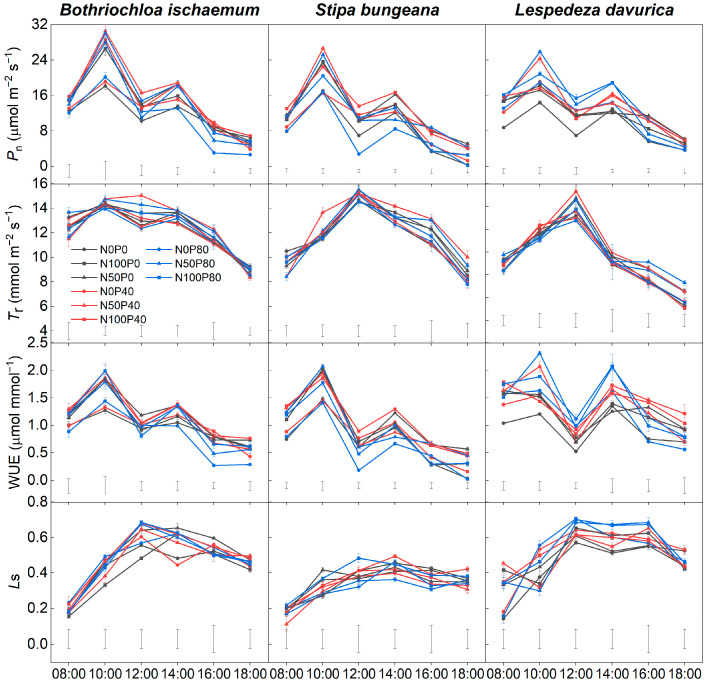
Diurnal changes of net photosynthetic rate (*P*_n_), transpiration rate (*T*_r_), instantaneous water use efficiency (WUE), and stomatal limitation value (*L*_s_) of the three species under different N and P addition treatments. Vertical bars indicate LSD values.

The diurnal changes of net photosynthetic rate (*P*_n_) and leaf instantaneous water use efficiency (WUE) of the three dominant species showed a double-peak curve under different N and P addition treatments. The first peak appeared at 10:00 h, the second at 14:00 h, and the midday depression of the photosynthesis (so-called “noon break”) appeared at around 12:00 h (Figure 2). The leaf transpiration rate (*T*_r_) of *B. ischaemum* mostly showed a double-peak diurnal course. While diurnal changes of *T*_r_ in *S. bungeana* and *L. davurica* showed a single peak.

Compared with CK (i.e., N0P0), N addition alone and addition of N and P combined significantly increased the *P*_n_ values at 10:00 h and 14:00 h in the three species (except under N50P40 and N50P80 treatments in *S. bungeana*). The greatest *P*_n_ values appeared at 10:00 h under N and P combined treatments (i.e., N50P40 and N50P80) for the three species. The WUE of the three species significantly increased by N addition alone compared with CK (Table 1). The WUE of *L. davurica* increased significantly under all levels of P alone additions, while the WUE of the two grasses only significantly increased under N0P40 (Table 1). Under N and P combined addition, the maximum WUE values of *B. ischaemum*, *S. bungeana,* and *L. davurica* were 1.17, 1.09, and 1.47 μmol mmol^−1^, respectively (Table 1).

N addition, alone, significantly increased the *L*_s_ values of *B. ischaemum* and *L. davurica* (Table 1). P addition, alone, increased (*p* < 0.05) and decreased (*p* < 0.05) the *L*_s_ of *L davurica* and *S. bungeana*, respectively, while only significantly increasing the *L*_s_ of *B. ischaemum* under N0P80 treatment. N and P interaction significantly affected the *L*_s_ values of the three species (Table 1). Under the addition of N50 combined with P, the *L*_s_ of *L. davurica* increased, and those of *B. ischaemum* decreased (both *p* < 0.05). Under the addition of N100 combined with P, the *L*_s_ of *B. ischaemum* significantly increased, while those of *S. bungeana* decreased significantly (*p* < 0.05; Table 1).

**Figure 3:**
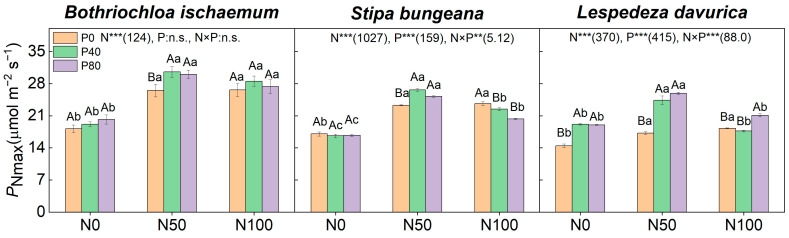
Leaf maximum net photosynthetic rate (*P*_Nmax_) of the three species under different N and P addition treatments. Values are mean ± SD. Different capital letters above the column indicate significant differences among P additions under each N addition rate, while different small letters indicate significant differences among N additions under each P addition rate. Numbers in parentheses are *F*-values, while ‘**’, and ‘***’ indicate *p* ≤ 0.01, and *p* ≤ 0.001, respectively. ‘n.s.’ indicates no significant difference.

N addition, alone, increased the *P*_Nmax_ values of the three species (*p* < 0.05), while there was no difference between them under N50 and N100 (Figure 3). The increase in *P*_Nmax_ in the two grasses was about two times larger than those of *L. davurica* under N addition alone (Figure 3). P addition, alone, increased the *P*_Nmax_ values of *L. davurica* and *S. bungeana* (*p* < 0.05; Figure 3). N and P combined addition only significantly affected the *P*_Nmax_ values of *S. bungeana* and *L. davurica* (*p* < 0.05; Figure 3).

### 2.3. Leaf SPAD Value

**Figure 4:**
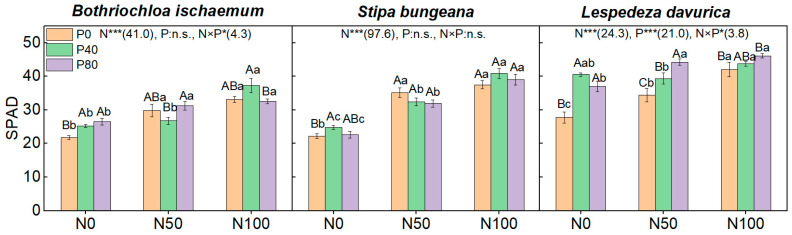
Leaf SPAD values of the three species under different N and P addition treatments. Different capital letters above the column indicate significant differences among P additions under each N addition rate, while different small letters indicate significant differences among N additions under each P addition rate. Numbers in parentheses are *F*-values, while ‘*’ and ‘***’ indicate *p* ≤ 0.05 and *p* ≤ 0.001, respectively. ‘n.s.’ indicates no significant difference.

N addition, alone, significantly increased the SPAD values of the three species (Figure 4). P addition, alone, significantly increased the SPAD values of *B. ischaemum* and *L. davurica* (*p* < 0.05), while SPAD values only increased under low levels of P addition alone (i.e., N0P40) in *S. bungeana* (*p* < 0.05). Addition of N and P combined significantly increased the SPAD values of both *B. ischaemum* and *L. davurica* (Figure 4).

### 2.4. Leaf N and P Concentration (N_mass_ and P_mass_) and N_mass_/P_mass_ Ratio

**Figure 5:**
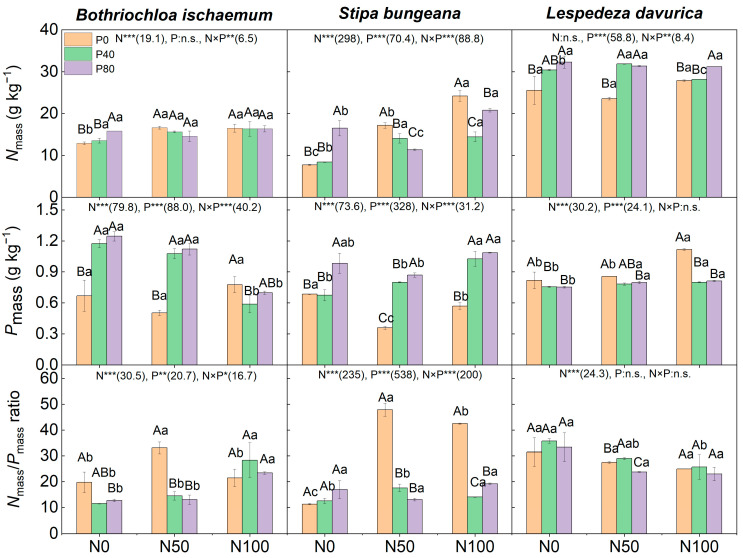
Leaf nitrogen (N) and phosphorus (P) content (*N*_mass_ and *P_mass_*), as well as the *N*_mass_/*P_mass_* ratio, of the three species under different N and P addition treatments. Values are mean ± SD. Different capital letters above the column indicate significant differences among P additions under each N addition rate, while different small letters indicate significant differences among N additions under each P addition rate. Numbers in parentheses are *F*-values, while ‘*’, ‘**’, and ‘***’ indicate *p* ≤ 0.05, *p* ≤ 0.01, and *p*≤ 0.001, respectively. ‘n.s.’ indicates no significant difference.

N addition, alone, significantly increased the *N_mass_* values of the two grasses, but it had no effects on *N_mass_* of *L. davurica*. The high level of P addition, alone (N0P80), significantly increased *P_mass_* of all species except *L. davurica* (Figure 5). N and P interaction significantly affected *N_mass_* of the three species, while it only significantly affected *P_mass_* of the two grasses. Under the addition of N50 combined with P, the *P_mass_* of *B. ischaemum* and *S. bungeana*, as well as the *N_mass_* of *L. davurica*, increased significantly. Under the addition of N100 combined with P, the *P_mass_* of *S. bungeana* increased significantly, while those of *L. davurica* decreased significantly (Figure 5).

N addition, alone, significantly affected the *N_mass_/P_mass_* ratios of all three species. The low level of N addition alone (N50P0) significantly increased the *N_mass_/P_mass_* of the two grasses; the high level of N addition, alone (N100P0), only increased the *N_mass_/P_mass_* of *S. bungeana* grass (*p* < 0.05, Figure 5). N and P addition interaction significantly affects the *N_mass_/P_mass_* of the two grasses but has no effect on the subshrub. Under the addition of N50 combined with P, the *N_mass_/ P_mass_* of *B. ischaemum* and *S. bungeana* decreased significantly, while the *N_mass_/P_mass_* of *L. davurica* increased under N50P40 treatment (*p* < 0.05). Under the addition of N100 combined with P, the *N_mass_/ P_mass_* of *S. bungeana* decreased significantly, while the *N_mass_/P_mass_* of *B. ischaemum* and *L. davurica* had no significant changes (Figure 5).

### 2.5. Specific Leaf Area (SLA) and Leaf Dry Matter Content (LDMC)

**Figure 6:**
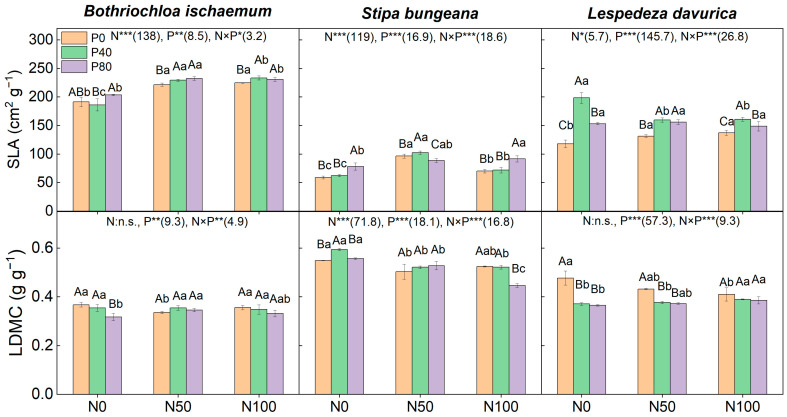
Specific leaf area (SLA) and leaf dry matter content (LDMC) of the three species under different N and P addition treatments. Different capital letters above the column indicate significant differences among P additions under each N addition rate, while different small letters indicate significant differences among N additions under each P addition rate. Numbers in parentheses are *F*-values, while ‘*’, ‘**’, and ‘***’ indicate *p* ≤ 0.05, *p* ≤ 0.01, and *p* ≤ 0.001, respectively. ‘n.s.’ indicates no significant difference.

N addition, alone, significantly increased the SLA values of the three species, and the low level of N addition alone (N50P0) decreased the LDMC of the two grasses (*p* < 0.05, Figure 6). P addition, alone, significantly increased SLA of *L. davurica*, while it had limited effects on the two grasses (Figure 6). N and P addition interaction significantly affected both the SLA and LDMC values of all three species. Under the addition of N50 combined with P, the SLA of *B. ischaemum* and *L. davurica* increased significantly (*p* < 0.05), and the SLA of *S. bungeana* increased under N50P40, while it decreased significantly under N50P80 (*p* < 0.05); LDMC was comparable between different levels of P additions in the two grasses, but it decreased in *L. davurica*. Under the addition of N100 combined with P, the SLA values of *B. ischaemum* and *L. davurica* increased significantly, and the SLA of *S. bungeana* increased (*p* < 0.05) only under the high level of N addition (N100P80); LDMC, among different levels of P addition (i.e., N100P40 and N100P80), was comparable in *B. ischaemum* and *L. davurica*, while it significantly decreased under N100P80 in *S. bungeana* (Figure 6).

**Figure 7:**
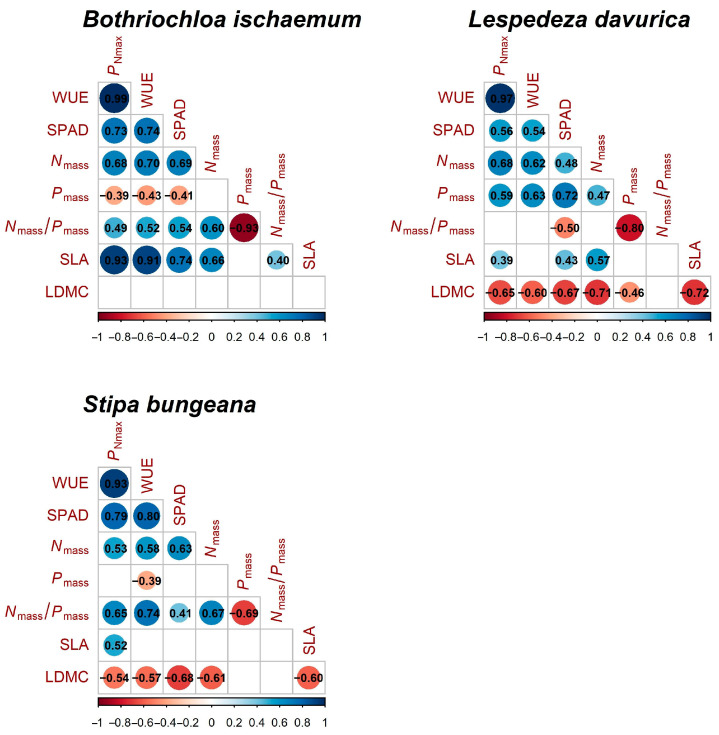
Heatmap of Pearson correlation testing relationships between *P*_Nmax_, WUE, and leaf functional traits (*N*_mass_, *P*_mass_, SLA, and LDMC) in the three species. Numbers in circles indicate the Pearson correlation coefficients.

**Figure 8:**
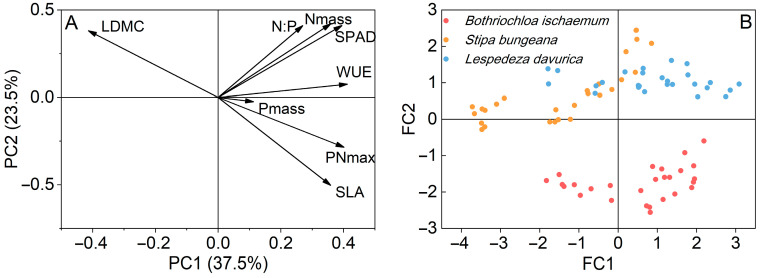
Principal component analysis (PCA) of photosynthetic characteristics (*P*_Nmax_ and WUE) and leaf functional traits (SPAD, *N*_mass_, *P*_mass_, SLA, and LDMC) in the three species under N and P additions. (**A**) Loadings for each leaf trait; (**B**) Factor scores for each species.

**Figure 9:**
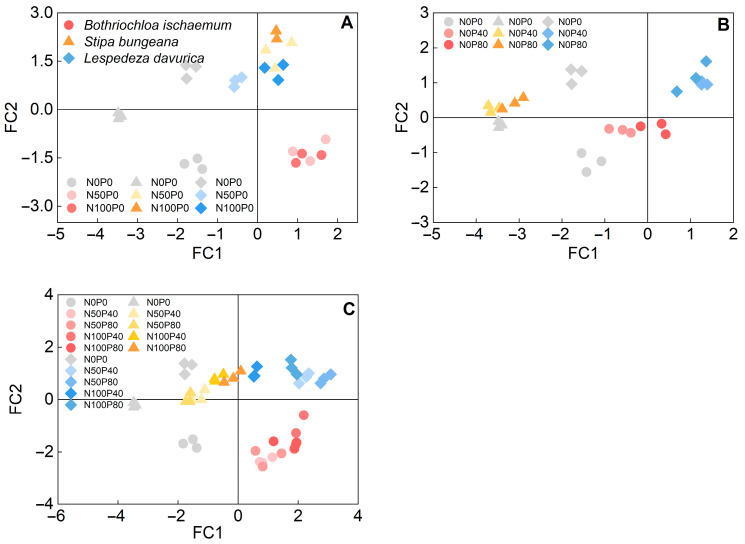
Score plots of PCA analysis of photosynthetic characteristics (*P*_Nmax_ and WUE) and leaf economic traits (SPAD, N_mass_, P_mass_, SLA, and LDMC) in the three species under N and P additions. (**A**) N addition alone; (**B**) P addition alone; (**C**) N and P combined addition.

*P*_Nmax_ was positively correlated with WUE, SPAD, *N*_mass_, and SLA in all three species, while it was negatively correlated with LDMC in *S. bungeana* and *L. davurica* (Figure 7). PCA analysis showed that the variance explained by the first and second principal components was 37.5% and 23.5%, respectively, with a total value of 61% (Figure 8). The first principal component had a high correlation with *P*_Nmax_, WUE, *P*_mass_, and SLA; the second principal component had high correlation with LDMC. SPAD, *N*_mass_, and *N_mass_/ P_mass_* ratio are correlated with both principal components (Figure 8). In a score plot of PCA analysis, under N addition alone, *B. ischaemum* and *L. davurica* gradually moved to the right with N addition level, and *S. bungeana* gradually moved to the upper right. Under P addition, alone, *L. davurica* gradually moved to the right with P addition level, and *B. ischaemum* slightly moved to the upper right. Under the addition of N combined with P, with the increase in fertilizer application, all three species moved towards higher *P*_Nmax_, WUE, SPAD, and SLA values (Figure 9).

## 3. Discussion

The diurnal dynamics of photosynthesis reflect plants’ sustained ability to carry out physiological metabolism and biomass accumulation throughout the daytime [45,46], which have been extensively studied in numerous dryland species (e.g., [19,20,21,22]). Our results, corroborated with others, showed that the diurnal course of photosynthetic rate showed a double-peaked curve in the three grassland dominant species, and they showed an evident “noon break” of photosynthesis. The noon break is a mechanism to avoid stresses such as excess light, high temperature, and water deficit during the midday [47], and it is a result of stomatal or non-stomatal restriction [48]. Our measurements showed that, during the period of 10:00–12:00 h, the *P*_n_ values gradually decreased while the *L*_s_ values increased (Figure 2 and Table 1), suggesting the “noon break” was likely caused by stomatal limitation [46,48]. In line with our first hypothesis and consistent with others (e.g., [49,50]), the *P*_n_ values of the three species considerably increased by nutrient addition, particularly during the peak photosynthetic period (~10:00 h). This is expected since environmental conditions (e.g., light and temperature) are relatively optimal for photosynthesis during this period, hence the promotion of nutrient addition would be most effective. In addition, during the noon time with high air temperature and light radiation, the *P_n_* was slightly increased after fertilization, which may be due to increased stomatal conductance due to N and P addition, and this ostensibly alleviated the “noon break” [51]. Our study indicated that N and P fertilization could improve the photosynthetic ability of the three species at the diurnal scale and increase the daily accumulative carbon assimilation. However, we only focused on short-term responses during the peak growing season (i.e., July), so future studies should be taken to further assess intra- or interannual patterns of their photosynthesis to better understand the long-term effects of fertilization.

Both N and P are essential elements of key compounds involved in the photosynthetic process, and appropriate N and P additions would increase the content of these compounds and, subsequently, the photosynthetic rate [17,26,30]. This was observed in our study: the *P*_Nmax_, SPAD, and *N*_mass_ of the three species increased significantly after N addition alone, and there were strong positive correlations between *N*_mass_, SPAD, and *P*_Nmax_ (*p <* 0.05; Figure 3, Figure 4 and Figure 5 and Figure 7). The increase in *P*_Nmax_ and WUE with the N addition level was much greater (larger regression slopes) in the two grass species than in the legume *L. davurica* (Table 2), with the greatest increase (the largest slope) of *P*_Nmax_ and WUE, along with the N addition level, in C_4_ grass *B. ischaemum* (Table 2). Together, this suggested that the two grasses were more sensitive to N addition alone than the legume. This is consistent with our previous study quantifying the plant biomass of *B. ischaemum* and *L. davurica* mixtures under varying soil moisture and nutrient supplies [52]. We suspect that the subshrub *L. davurica* may not be N-limited due to its N fixation ability and is, thereby, insensitive to exogenous N fertilization. On the other hand, the photosynthetic rate does not continuously increase with N addition amounts after passing a threshold [29,53], which was also observed, here, as the *P*_Nmax_ values of the three species were not significantly different between N50 and N100 (Figure 3 and Figure 6; Table 1). Fossil fuel combustion and extensive fertilization have greatly increased atmospheric N deposition globally in recent decades [54]. Chronic N input by long-term N deposition may, hence, alleviate N limitation and promote plant photosynthesis and growth of regional grassland species, but it may, meanwhile, intensify plant P limitation by increasing P demand [55,56].

Here, the P addition, alone, had greater effects on *P*_Nmax_, SPAD, and SLA of the leguminous *L. davurica* among the three species (Figure 3, Figure 4 and Figure 6; Table 2). This may be ascribed to P, as it could promote the activity of nitrogenase in the root nodules of legumes and enhance their photosynthesis [34,57], and elevated leaf P content can also directly improve photosynthetic capacity by promoting ATP and NADPH synthesis, as well as regeneration of RuBP [33,58]. Compared with N or P addition alone, the three species had higher *P*_Nmax_, WUE, and SLA values under N and P combined additions, suggesting a synergetic effect of N and P on plant photosynthesis (Figure 5 and Figure 6). This confirms our second hypothesis and suggests that appropriate N and P combined fertilization should be considered to maintain regional grassland productivity. A myriad of studies have documented this synergetic effect in grasslands worldwide (e.g., [59,60]). Previous studies in the Loess Plateau grasslands also reported the N and P combination had synergetic effects on community productivity [44]. A recent long-term (over 66 years) nutrient addition study in a mesic grassland in South Africa also highlighted that N and P combined addition promoted plant P acquisition and uptake (e.g., increased organic P storage, P recycling, and plant P utilization), which may contribute to the synergetic effect of N and P combined addition [59].

Drylands (e.g., the semiarid Loess Plateau) are often co-limited by water and nutrients [8], as well as characterized by frequent drought events, which greatly impact plant N and P uptake [61]. Nutrient addition, such as N, at an appropriate rate could improve post-drought recovery of grassland and increase the aboveground biomass production [62]. Contrarily, some studies reported that nutrient addition increased grassland drought sensitivity and constrained its recovery from drought events [10]. Besides, grass species with different photosynthetic pathways (C_3_ vs. C_4_) may respond differentially to drought and rewatering under nutrient addition conditions [63]. For the regional grassland, previous studies have quantified the photosynthetic responses of dominant species following rainfall events and reported species-specific patterns [64]. Nevertheless, the interaction of soil moisture (especially drought) and fertilization on dominant species performance remains less understood in the regional grassland and should be assessed, considering recurrent drought events, under future climate scenarios [65].

Leaf functional traits, particularly those so-called economic traits, are invoked to explain plant resource acquisition and utilization [36]. Among them, the leaf *N_mass_/ P_mass_* ratio indicates environmental N and P availability where the plant grows [56]. In general, *N_mass_/ P_mass_* ratio less than 10 indicates the N limitation, and greater than 20 indicates the P limitation [56]. The leaf *N_mass_/ P_mass_* ratio of the three species, averaged across treatments, was 18.8 (*B. ischaemum*), 11.3 (*S. bungeana*), and 24.3 (*L. davurica*), respectively (Figure 5), suggesting species-specific N and P limitations. The *N_mass_/P_mass_* ratio of the two grasses increased significantly with N addition, while no noticeable change was found in *L. davurica* (Figure 5). This indicates that N addition may lead to P limitation in the two grass species. Meanwhile, increased soil N and P availability would release plants from nutrient competition to other resource competition, such as light and water [66]. Grassland dominant species may accordingly alter their leaf functional traits to maximize light harvesting to maintain dominance. According to the LES theory, plants with higher light capture, resource acquisition, and turnover capacity show higher SLA, *N*_mass_, and *P*_mass_ in contrast to the slow-growth ones with higher LDMC and conservative nutrient resource use [36]. Similar to other studies (e.g., [66]), the three species studied here shifted to a fast-growth strategy after N addition with larger, thinner, and N-rich leaves (higher SLA, *N*_mass,_ and SPAD), as well as higher assimilation rate per unit leaf area (higher *P*_Nmax_). Though score plots from PCA analysis indicated that, under N addition, three species adopted different strategies to improve their light harvesting: C_4_ grass *B. ischaemum* mainly by increasing SLA and *P*_Nmax_, while C_3_ grass *S. bungeana* and C_3_ subshrub *L. davurica* primarily increased leaf N content and SPAD (Figure 9), and only *L. davurica* had notable shifts in photosynthetic and leaf functional traits under P additions (Figure 9B), which suggests that the three species had different trade-off strategies in photosynthetic performance and leaf economic traits in response to N and/or P addition [17,66], and these should be considered when assessing N and P fertilization effects on community structure and functions. The *P*_Nmax_ values of the three species were mostly highest under the ‘N50P40′ treatment among all treatments, indicating that it could be considered an optimal fertilization measure for improving grassland production.

## 4. Materials and Methods

### 4.1. Site Description

This work was conducted at the Zhifanggou watershed (109°13’46’’–109°16’03’’ E, 36°42’42’’–36°46’28’’ N), located in the Ansai District, Yan’an City, Shaanxi Province, China. It has a semiarid continental monsoon climate. The mean annual temperature is 8.8 °C, with the lowest temperature being −6.9 °C in January and the highest being 22.6 °C in July. The mean annual rainfall is 507 mm. The soil is classified as Calcaric Cambisol. Rainfall shows a highly seasonal variability with ca. 82% occurring from May to September (the growing season). The soil available N, P, and K were 20.9–71.3 mg kg^−1^, 1.6–2.8 mg kg^−1^, and 10.07–30.97 g kg^−1^, respectively, and soil pH was 8.4–8.8 [67]. The targeted grassland is dominated by xerophytic plants, e.g., *B. ischaemum*, *S. bungeana*, *L. davurica*, *Artemisia sacrorum*, and *Artemisia giraldii*.

### 4.2. N and P addition

A grassland community (20 × 30 m) was fenced to exclude grazing since May 2017. A randomized split-plot design with three N addition rates at the main plot level and three P addition rates at the subplot level was carried out. The main plot was 4 × 4 m, and N addition rates were N0 (0 kg N), N50 (50 kg N ha^−1^ yr^−1^), and N100 (100 kg N ha^−1^ yr^−1^). The N50 and N100 treatments were about 2 and 4 times the annual average N deposition rate in the loess hilly area [~21.76 kg (N) ha^−1^ yr^−1^] [68]. N was applied as calcium ammonium nitrate [5Ca(NO_3_)_2_ NH_4_NO_3_ 10H_2_O] (15.5% of N). Each main plot was divided into four subplots (2 × 2 m). P was applied as triple superphosphate [Ca(H_2_PO_4_)_2_·H_2_O] (45% of P), and the addition rates were set to 0, 1, and 2 times the local fertilization rate, corresponding to P0 (0 kg P_2_O_5_), P40 (40 kg P_2_O_5_ ha^−1^ yr^−1^), and P80 (80 kg P_2_O_5_ ha^−1^ yr^−1^) [44].

Totally, there were 9 treatments, including a control (N0P0), two N addition alone treatments (N50P0, N100P0), two P addition alone treatments (N0P40, N0P80), four N and P combined addition treatments (N50P40, N50P80, N100P40, N100P80), and three replicates per treatment. N and P additions were conducted once a year, on rainy days, from 2017–2019 (4 June 2017, 21 May 2018, and 13 June 2019).

### 4.3. Ecophysiological Measurements

#### 4.3.1. Diurnal Variations of Photosynthesis

The portable photosynthesis system (CIRAS-2, PP Systems, Amesbury, MA, USA) was used to measure the diurnal changes of photosynthesis of *B. ischaemum, S. bungeana*, and *L. davurica*, successively, and all measurements were conducted on three consecutive sunny days from 20–22 July 2019 (one species per day). The measurement was taken on one newly fully-expanded healthy leaf per species per treatment from 8:00–18:00 h with 2 h intervals. The measured parameters include net photosynthetic rate (*P*_n_, μmol·m^−2^·s^−1^), transpiration rate (*T*_r_, mmol·m^−2^·s^−1^), intercellular CO_2_ concentration (*C*_i_, μmol·mol^−1^), and environmental factors, including photosynthetically active radiation (PAR, μmol·m^−2^·s^−1^), air temperature (*T*_a_, °C), and relative humidity (RH, %). The photosynthetic rate at 10:00 h was taken as the maximum net photosynthetic rate (*P*_Nmax_, μmol·m^−2^·s^−1^). Instantaneous water use efficiency (WUE, μmol mmol^−1^) was calculated as *P*_n_/*T*_r_. Stomatal limitation value (*L*_s_) was derived by 1−*C*_i_/*C*_a_ [48].

#### 4.3.2. Leaf SPAD Value

Leaf SPAD value (a measure of leaf relative chlorophyll content) was measured on three newly fully-expanded healthy leaves per species per treatment using a chlorophyll meter (SPAD-502 model, Konica-Minolta, Osaka, Japan) on 20–22 July 2019.

#### 4.3.3. Leaf Functional Traits

The 10–20 newly-fully expanded healthy leaves were randomly sampled per species per treatment, stored in zipped plastic bags, and quickly taken back to the laboratory, in an insulated box with ice packs, for leaf functional traits measurements. Leaves were weighed with an analytical balance (d = 0.0001 g). The fresh leaves were scanned (Epson duplex scanner, Epson, Tokyo, Japan), and the leaf area was derived using ImageJ (National Institutes of Health, Bethesda, MD, USA). Then, leaves were oven-dried at 75 °C for 24 h and ground with a high-throughput tissue grinder (MM-400, Retsch, Haan, Germany). Specific leaf area (SLA, m^2^ g^–1^) was calculated as leaf area divided by leaf dry mass. Leaf dry matter content (LDMC, g g^–1^) was calculated as leaf dry mass divided by fresh mass. After digestion with H_2_SO_4_-HClO_4_, the mass-based leaf N concentration (*N*_mass_) was obtained using a Kjeldahl N analyzer (FOSS-8400, Foss, Höganäs, Denmark). The mass-based leaf P concentration *(P*_mass_) was determined by a molybdenum blue colorimetry (UV-2600 ultraviolet-visible spectrophotometer, Shimadzu, Kyoto, Japan). *N_mass_/P_mass_* ratio was then calculated.

### 4.4. Statistical Analysis

All statistical analyses were performed with SPSS 20.0. One-way analysis of variance (ANOVA) was used to compare the differences in leaf photosynthetic characteristics (WUE, *L*_s_, and *P*_Nmax_) and leaf functional traits (SPAD, *N*_mass_, *P*_mass_, *N*_mass_/*P*_mass_, SLA, and LDMC) of the three species under different N and P addition treatments. Tukey’s HSD test was used for multiple comparisons. Two-way ANOVA was used to test the effects of N addition, P addition, and their interaction on *P*_Nmax_, SPAD value, *N*_mass_, *P*_mass,_ SLA, and LDMC. Pearson correlation was used to explore the relationship between leaf photosynthetic characteristics (*P*_Nmax_, WUE) and leaf functional traits (SPAD value, *N*_mass_, *P*_mass,_ *N*_mass_/ *P*_mass_, SLA, and LDMC). Multiple linear regression was used to explore the relationship between N addition, P addition, and their interaction, as well as *P*_Nmax_ and WUE. Principal component analysis (PCA) was conducted on photosynthetic characteristics and leaf functional traits. Graphing was performed with Origin 2021 (Origin Lab Software, Chicago, IL, USA).

## 5. Conclusions

Our three-year field fertilization study suggested that N addition—alone or combined with P—improved the photosynthesis of the three grassland dominant species on the semiarid Loess Plateau of China. All three species shifted to a fast-growth strategy with increased *P*_Nmax_, SLA, and *N*_mass_, as well as reduced LDMC under N and/or P addition. Furthermore, species-specific shifts in leaf functional traits were observed among the three species following N and/or P addition, of which C_4_ grass *B. ischaemum* increased SLA and *P*_Nmax_, and C_3_ grass *S. bungeana* and subshrub *L. davurica* mainly increased leaf N and SPAD. P addition seems to only effectively impact the *P_n_* of *L. davurica*. Evident N and P synergetic effects on the photosynthetic performance in all three species were observed, and a combination of 50 kg ha^−1^ yr^−1^ N and 40 kg ha^−1^ yr^−1^ P addition could be considered optimal fertilization for improving grassland productivity locally.

## Figures and Tables

**Table 1:** Instantaneous water use efficiency (WUE) and stomatal limitation value (*L*_s_) of the three species under different N and P additions (mean ± SD, *n* = 3).

Species	Treatment	WUE (μmol mmol^–1^)			*L*_s_		
		P0	P40	P80	P0	P40	P80
*B. ischaemum*	N0	0.95 ± 0.02 ^B,b^	0.97 ± 0.02 ^A,b^	0.81 ± 0.01 ^C,b^	0.44 ± 0.00 ^B,c^	0.45 ± 0.01 ^B,b^	0.48 ± 0.00 ^A,a^
	N50	1.15 ± 0.02 ^A,a^	1.17 ± 0.01 ^A,a^	1.10 ± 0.01 ^B,a^	0.50 ± 0.01 ^A,a^	0.46 ± 0.01 ^B,b^	0.48 ± 0.01 ^B,a^
	N100	1.10 ± 0.05 ^A,a^	1.21 ± 0.02 ^A,a^	1.11 ± 0.04 ^A,a^	0.42 ± 0.00 ^B,b^	0.50 ± 0.01 ^A,a^	0.48 ± 0.01 ^A,a^
*S. bungeana*	N0	0.69 ± 0.01 ^B,b^	0.78 ± 0.01 ^A,b^	0.59 ± 0.01 ^C,b^	0.68 ± 0.00 ^A,a^	0.35 ± 0.01 ^B,a^	0.33 ± 0.01 ^B,b^
	N50	1.05 ± 0.01 ^A,a^	0.97 ± 0.02 ^B,a^	0.97 ± 0.01 ^B,a^	0.36 ± 0.02 ^A,c^	0.35 ± 0.01 ^A,a^	0.32 ± 0.01 ^B,b^
	N100	0.91 ± 0.02 ^A,a^	1.09 ± 0.02 ^A,a^	0.84 ± 0.02 ^B,a^	0.57 ± 0.03 ^A,b^	0.36 ± 0.00 ^B,a^	0.37 ± 0.00 ^B,a^
*L. davurica*	N0	0.93 ± 0.01 ^C,b^	1.36 ± 0.03 ^A,a^	1.18 ± 0.01 ^B,b^	0.45 ± 0.01 ^B,b^	0.52 ± 0.01 ^A,a^	0.51 ± 0.01 ^A,b^
	N50	1.24 ± 0.00 ^C,a^	1.33 ± 0.02 ^B,b^	1.47 ± 0.06 ^A,a^	0.48 ± 0.01 ^B,a^	0.50 ± 0.00 ^A,a^	0.52 ± 0.01 ^A,b^
	N100	1.22 ± 0.02 ^B,a^	1.36 ± 0.01 ^A,a^	1.43 ± 0.03 ^A,a^	0.51 ± 0.00 ^B,a^	0.51 ± 0.02 ^B,a^	0.55 ± 0.01 ^A,a^
*B. ischaemum*		N ***(269); P ***(47.9); N × P ***(8.4)			N ***(23.3); P ***(36.6); N × P ***(63.2)		
*S. bungeana*		N ***(1084); P ***(223); N × P ***(75.5)			N ***(66.6); P **(8.62); N × P ***(14.1)		
*L. davurica*		N ***(156); P ***(223); N × P ***(46.8)			N ***(26.4); P ***(45.8); N × P ***(8.93)		

Data with different capital letters indicate significant differences among P additions under each N addition rate, while different small letters indicate significant differences among N additions under each P addition rate. Numbers in parentheses are *F*-values, while ‘**’ and ‘***’ indicate *p* ≤ 0.01, and *p* ≤ 0.001, respectively.

**Table 2:** Regression slopes (SE) derived from the multiple linear regression analysis between photosynthetic characteristics (*P*_Nmax_ and WUE) and N addition, P addition, and N and P addition interaction in the three species.

Species	Variable	*P*_Nmax_	WUE
	N	0.0894 (0.0161) ***	0.0060 (0.0010) ***
*B. ischaemum*	P	0.0182 (0.0201) ^n.s.^	0.0012 (0.0013) ^n.s.^
	N × P	−0.0001 (0.0003) ^n.s.^	0.0000 (0.0000) ^n.s.^
	N	0.0640 (0.0131) ***	0.0049 (0.0009) ***
*S. bungeana*	P	−0.0250 (0.0164) ^n.s.^	−0.0029 (0.0012) *
	N × P	−0.0002 (0.0003) ^n.s.^	−0.0001 (0.0001) ^n.s.^
	N	0.0238 (0.0205) ^n.s.^	0.0022 (0.0019) ^n.s.^
*L. davurica*	P	0.0782 (0.0257) **	0.0071 (0.0024) **
	N × P	−0.0002 (0.0004) ^n.s.^	0.0000 (0.0000) ^n.s.^

‘^n.s.^’, ‘*’, ‘**’, and ‘***’ indicate *p* > 0.05, *p* ≤ 0.05, *p* ≤ 0.01, and *p* ≤ 0.001, respectively. Significant slopes are in bold.

## Data Availability

The data presented in this study are available on reasonable request from the corresponding author.

## References

[1] Huang L., Shao M. (2019). Advances and perspectives on soil water research in China’s Loess Plateau. Earth Sci. Rev..

[2] Gang C.C., Zhao W., Zhao T., Zhang Y., Gao X.R., Wen Z.G. (2018). The impacts of land conversion and management measures on the grassland net primary productivity over the Loess Plateau, Northern China. Sci. Total Environ..

[3] Sun C.J., Hou H.X., Chen W. (2021). Effects of vegetation cover and slope on soil erosion in the Eastern Chinese Loess Plateau under different rainfall regimes. Peer J..

[4] Yang Y., Liu H., Yang X., Yao H.J., Deng X.Q., Wang Y.Q., An S.S., Kuzyakov Y., Chang S.X. (2022). Plant and soil elemental C:N:P ratios are linked to soil microbial diversity during grassland restoration on the Loess Plateau, China. Sci. Total Environ..

[5] Bai Y., Wu J., Clark C.M., Naeem S., Pan Q., Huang J., Zhang L., Han X. (2010). Tradeoffs and thresholds in the effects of nitrogen addition on biodiversity and ecosystem functioning: Evidence from inner Mongolia Grasslands. Glob. Change Biol..

[6] Wang Y., Sun Y., Chang S., Wang Z., Fu H., Zhang W., Hou F. (2020). Restoration practices affect alpine meadow ecosystem coupling and functions. Rangel. Ecol. Manag..

[7] Botter M., Zeeman M., Burlando P., Fatichi S. (2021). Impacts of fertilization on grassland productivity and water quality across the European Alps under current and warming climate: Insights from a mechanistic model. Biogeosciences.

[8] Bharath S., Borer E.T., Biederman L.A., Blumenthal D.M., Fay P.A., Gherardi L.A., Knops J.M.H., Leakey A.D.B., Yahdjian L., Seabloom E.W. (2020). Nutrient addition increases grassland sensitivity to droughts. Ecology.

[9] Smith V.H., Schindler D.W. (2009). Eutrophication science: Where do we go from here?. Trends Ecol. Evol..

[10] Meng B., Li J., Maurer G.E., Zhong S., Yao Y., Yang X., Collins S.L., Sun W. (2021). Nitrogen addition amplifies the nonlinear drought response of grassland productivity to extended growing-season droughts. Ecology.

[11] Suding K.N., Collins S.L., Gough L., Clark C., Cleland E.E., Gross K.L., Milchunas D.G., Pennings S. (2005). Functional-and abundance-based mechanisms explain diversity loss due to N fertilization. Proc. Natl. Acad. Sci. USA.

[12] Socher S.A., Prati D., Boch S., Müller J., Klaus V.H., Hölzel N., Fischer M. (2012). Direct and productivity-mediated indirect effects of fertilization, mowing and grazing on grassland species richness. J. Ecol..

[13] Harpole W.S., Sullivan L.L., Lind E.M., Firn J., Adler P.B., Borer E.T., Chase J., Fay P.A., Hautier Y., Hillebrand H. (2016). Addition of multiple limiting resources reduces grassland diversity. Nature.

[14] Hautier Y., Zhang P.F., Loreau M., Wilcox K.R., Seabloom E.W., Borer E.T., Wang S. (2020). General destabilizing effects of eutrophication on grassland productivity at multiple spatial scales. Nat. Commun..

[15] Liu Z.P., Shao M.A., Wang Y.Q. (2013). Spatial patterns of soil total nitrogen and soil total phosphorus across the entire Loess Plateau region of China. Geoderma.

[16] Zhang L., Wei X., Hao M., Zhang M. (2015). Changes in aggregate-associated organic carbon and nitrogen after 27 years of fertilization in a dryland alfalfa grassland on the loess plateau of china. J. Arid Land.

[17] Chen Z.F., Xiong P.F., Zhou J.J., Lai S.B., Jian C.X., Wang Z., Xu B.C. (2020). Photosynthesis and nutrient-use efficiency in response to N and P addition in three dominant grassland species on the semiarid Loess Plateau. Photosynthetica.

[18] Qu Q., Wang M., Xu H., Yan Z., Liu G., Xue S. (2022). Role of soil biotic and abiotic properties in plant community composition in response to nitrogen addition. Land Degrad. Dev..

[19] Senock R.S., Devine D.L., Sisson W.B., Donart G.B. (1994). Ecophysiology of three C_4_ perennial grasses in the northern Chihuahuan Desert. Southwest. Nat..

[20] Nippert J.B., Fay P.A., Knapp A.K. (2007). Photosynthetic traits in C_3_ and C_4_ grassland species in mesocosm and field environments. Environ. Exp. Bot..

[21] Huxman T.E., Smith S.D. (2001). Photosynthesis in an invasive grass and native forb at elevated CO_2_ during an El Nino year in the Mojave Desert. Oecologia.

[22] Adam N.R., Owensby C.E., Ham J.M. (2000). The effect of CO_2_ enrichment on leaf photosynthetic rates and instantaneous water use efficiency of Andropogon gerardii in the tallgrass prairie. Photosynth. Res..

[23] Nijs I., Impens I., Van Hecke P. (1992). Diurnal changes in the response of canopy photosynthetic rate to elevated CO2 in a coupled temperature-light environment. Photosynth. Res..

[24] Haase P., Pugnaire F.I., Clark S.C., Incoll L.D. (1999). Environmental control of canopy dynamics and photosynthetic rate in the evergreen tussock grass *Stipa tenacissima*. Plant Ecol..

[25] Evans J.R., Terashima I. (1987). Effects of nitrogen nutrition on electron transport components and photosynthesis in spinach. Funct. Plant Biol..

[26] Sinha D., Tandon P.K. (2020). An Overview of Nitrogen, Phosphorus and Potassium: Key Players of Nutrition Process in Plants. Sustain. Solut. Elem. Defic. Excess Crop Plants.

[27] Wang D., Ling T.Q., Wang P.P., Fan J.Z., Wang H., Zhang Y.Q. (2018). Effects of 8-year nitrogen and phosphorus treatments on the ecophysiological traits of two key species on tibetan plateau. Front. Plant Sci..

[28] Lai S.B., Xu S., Jian C.X., Chen Z.F., Zhou J.J., Yang Q., Wang Z., Xu B.C. (2021). Leaf photosynthetic responses to nitrogen and phosphorus additions of dominant species in farm-withdrawn grassland in the loess hilly-gully region. Acta Ecol. Sin..

[29] Xing H., Zhou W., Wang C., Li L., Li X., Cui N., Hao W., Liu F., Wang Y. (2021). Excessive nitrogen application under moderate soil water deficit decreases photosynthesis, respiration, carbon gain and water use efficiency of maize. Plant Physiol. Biochem..

[30] Holford I.C.R. (1997). Soil phosphorus: Its measurement, and its uptake by plants. Aust. J. Soil Res..

[31] Carstensen A., Herdean A., Schmidt S.B., Sharma A., Spetea C., Pribil M., Husted S. (2018). The impacts of phosphorus deficiency on the photosynthetic electron transport chain. Plant Physiol..

[32] Chaudhary M.I., Adu-Gyamfi J.J., Saneoka H., Nguyen N.T., Suwa R., Kanai S., El-Shemy H.A., Lightfoot D.A., Fujita K. (2008). The effect of phosphorus deficiency on nutrient uptake, nitrogen fixation and photosynthetic rate in mashbean, mungbean and soybean. Acta Physio. Plant.

[33] Liu C., Wang Y., Pan K. (2015). Effects of phosphorus application on photosynthetic carbon and nitrogen metabolism, water use efficiency and growth of dwarf bamboo (*Fargesia rufa*) subjected to water deficit. Plant Physiol. Bioch..

[34] Suriyagoda L., Lambers H., Ryan M.H., Renton M. (2010). Effects of leaf development and phosphorus supply on the photosynthetic characteristics of perennial legume species with pasture potential: Modelling photosynthesis with leaf development. Funct. Plant Biol..

[35] Walker A.P., Beckerman A.P., Gu L.H., Kattge J., Cernusak L.A., Domingues T.F., Scales J.C., Wohlfahrt G., Wullschlrger S.D., Woodward F.L. (2015). The relationship of leaf photosynthetic traits–*V*_cmax_ and *J*_max_–to leaf nitrogen, leaf phosphorus, and specific leaf area: A meta-analysis and modeling study. Ecol. Evol..

[36] Wright I.J., Reich P.B., Westoby M., Ackerly D.D., Baruch Z., Bongers F., Cavender-Bares J., Chapin T., Cornelissen J.H.C., Diemer M. (2004). The worldwide leaf economics spectrum. Nature.

[37] Reich P.B. (2014). The world-wide ‘fast-slow’ plant economics spectrum: A traits manifesto. J. Ecol..

[38] Wright J.P., Sutton-Grier A. (2012). Does the leaf economic spectrum hold within local species pools across varying environmental conditions?. Funct. Ecol..

[39] Mao W., Li Y.L., Zhao X.Y., Zhang T.H., Liu X.P. (2016). Variations of leaf economic spectrum of eight dominant plant species in two successional stages under contrasting nutrient supply. Pol. J. Ecol..

[40] Sasaki T., Lauenroth W.K. (2011). Dominant species, rather than diversity, regulates temporal stability of plant communities. Oecologia.

[41] Avolio M.L., Forrestel E.J., Chang C.C., La Pierre K.J., Burghardt K.T., Smith M.D. (2019). Demystifying dominant species. New Phytol..

[42] Avolio M.L., Koerner S.E., La Pierre K.J., Wilcox K.R., Wilson G.W.T., Smith M.D., Collins S.L. (2014). Changes in plant community composition, not diversity, during a decade of nitrogen and phosphorus additions drive above-ground productivity in a tallgrass prairie. J. Ecol..

[43] Yang Y., Dou Y., An S.S. (2017). Environmental driving factors affecting plant biomass in natural grassland in the Loess Plateau, China. Ecol. Indic..

[44] Chen Z.F., Xiong P.F., Zhou J.J., Yang Q., Wang Z., Xu B.C. (2020). Grassland productivity and diversity changes in responses to N and P addition depend primarily on tall clonal and annual species in semiarid Loess Plateau. Ecol. Eng..

[45] Geiger D.R., Servaites J.C. (1994). Diurnal regulation of photosynthetic carbon metabolism in C3 plants. Annu. Rev. Plant Biol..

[46] Du Y.C., Nose A., Kondo A. (2008). Diurnal changes in photosynthesis in sugarcane leaves. I. carbon dioxide exchange rate, photosynthesic enzyme activities and metabolite levels relating to the C_4_ pathway and the calvin cycle. Plant Prod. Sci..

[47] Muraoka H., Tang Y., Terashima I., Koizumi H., Washitani I. (2000). Contributions of diffusional limitation, photoinhibition and photorespiration to midday depression of photosynthesis in *Arisaema heterophyllum* in natural high light. Plant Cell Environ..

[48] Farquhar G.D., Sharkey T.D. (1982). Stomatal conductance and photosynthesis. Annu. Rev. Plant Physiol..

[49] Wu F.Z., Bao W.K., Li F.L., Wu N. (2008). Effects of water stress and nitrogen supply on leaf gas exchange and fluorescence parameters of *Sophora davidii* seedlings. Photosynthetica.

[50] Peng Y., Li C., Fritschi F.B. (2014). Diurnal dynamics of maize leaf photosynthesis and carbohydrate concentrations in response to differential N availability. Environ. Exp. Bot..

[51] Zhao H.B., Qi L., Liu Y.G. (2010). Effects of combined application of nitrogen and phosphorus on diurnal variation of photosynthesis at grain-filling stage and grain yield of super high-yielding wheat. Chin. J. Appl. Ecol..

[52] Xu B.C., Xu W.Z., Wang Z., Chen Z.F., Palta J.A., Chen Y.L. (2018). Accumulation of N and P in the legume *Lespedeza davurica* in controlled mixtures with the grass *Bothriochloa ischaemum* under varying water and fertilization conditions. Front. Plant Sci..

[53] Tian Z.W., Liu X.X., Gu S.L., Yu J.H., Zhang L., Zhang W.W., Jiang D., Cao W.X., Dai T.B. (2018). Postponed and reduced basal nitrogen application improves nitrogen use efficiency and plant growth of winter wheat. J. Integr. Agric..

[54] Ackerman D., Millet D.B., Chen X. (2019). Global estimates of inorganic nitrogen deposition across four decades. Glob. Biogeochem. Cycles.

[55] Li Y., Niu S., Yu G. (2016). Aggravated phosphorus limitation on biomass production under increasing nitrogen loading: A meta-analysis. Global Change Biol..

[56] Güsewell S. (2004). N: P ratios in terrestrial plants: Variation and functional significance. New Phytol..

[57] Naeem M., Khan M.M.A., Moinuddin, Idrees M., Aftab T. (2010). Phosphorus ameliorates crop productivity, photosynthetic efficiency, nitrogen-fixation, activities of the enzymes and content of nutraceuticals of *Lablab purpureus* L. Sci. Hortic..

[58] Reich P.B., Oleksyn J., Wright I.J. (2009). Leaf phosphorus influences the photosynthesis–nitrogen relation: A cross-biome analysis of 314 species. Oecologia.

[59] Schleuss P.M., Widdig M., Heintz-Buschart A., Kirkman K., Spohn M. (2020). Interactions of nitrogen and phosphorus cycling promote P acquisition and explain synergistic plant-growth responses. Ecology.

[60] Elser J.J., Bracken M.E., Cleland E.E., Gruner D.S., Harpole W.S., Hillebrand H., Ngai J.T., Seabloom E.W., Shurin B.J., Smith J.E. (2007). Global analysis of nitrogen and phosphorus limitation of primary producers in freshwater, marine and terrestrial ecosystems. Ecol. Lett..

[61] Mariotte P., Cresswell T., Johansen M.P., Harrison J.J., Keitel C., Dijkstra F.A. (2020). Plant uptake of nitrogen and phosphorus among grassland species affected by drought along a soil available phosphorus gradient. Plant Soil.

[62] Kinugasa T., Tsunekawa A., Shinoda M. (2012). Increasing nitrogen deposition enhances post-drought recovery of grassland productivity in the Mongolian steppe. Oecologia.

[63] Zhong S.Z., Xu Y., Meng B., Loik M.E., Ma J.Y., Sun W. (2019). Nitrogen addition increases the sensitivity of photosynthesis to drought and re-watering differentially in C_3_ versus C_4_ grass species. Front. Plant Sci..

[64] Niu F.R., Duan D.P., Chen J., Xiong P.F., Zhang H., Wang Z., Xu B.C. (2016). Eco-physiological responses of dominant species to watering in a natural grassland community on the semi-arid Loess Plateau of China. Front. Plant Sci..

[65] Sun C.X., Huang G.H., Fan Y., Zhou X., Lu C., Wang X.Q. (2019). Drought occurring with hot extremes: Changes under future climate change on Loess Plateau, China. Earth’s Future.

[66] Wan H.W., Yang Y., Bai S.Q., Xu Y.H., Bai Y.F. (2008). Variations in leaf functional traits of six species along a nitrogen addition gradient in *Leymus chinensis* steppe in Inner Mongolia. J. Plant Ecol..

[67] Zhao W., Zhang R., Huang C.Q., Wang B.Q., Cao H., Koopal L.K., Tan W.F. (2016). Effect of different vegetation cover on the vertical distribution of soil organic and inorganic carbon in the Zhifanggou watershed on the Loess Plateau. Catena.

[68] Liang T., Tong Y.A., Xu W., Wei Y., Lin W., Pang Y., Liu F., Liu X.J. (2016). Atmospheric nitrogen deposition in the Loess area of China. Atmos. Pollut. Res..

